# Health workforce data needed to minimize inequities associated with health-worker migration

**DOI:** 10.2471/BLT.23.290028

**Published:** 2023-11-21

**Authors:** Margaret Walton-Roberts, Ivy L Bourgeault

**Affiliations:** a Department of Geography and Environmental Studies, Wilfrid Laurier University, 75 University Avenue West, Waterloo, Ontario N2L 3C5, Canada.; b School of Sociological and Anthropological Studies, University of Ottawa, Ottawa, Canada.

## Abstract

A persistent challenge with health-worker migration is the inequities it creates. To minimize these inequities, systems of global governance of health-worker migration have arisen which include various global codes of practice, agreements and reporting requirements. Reporting that is rigorous, open and transparent, and subject to scrutiny from the public, researchers, civil society organizations and other interested stakeholders, is important. One element of these codes and agreements with perhaps the greatest potential to deal with the impact of health-worker migration is more robust planning of the health workforce to address the goal of self-sufficiency. Open platforms for data sharing enable engagement of the public and stakeholders with data on the distribution and national origin of health workers, and reveal policy strengths and weaknesses related to health-workforce planning. We explore recent policies directed at reducing the inequities from health-worker migration. While many of the examples used focus on nurses and doctors, the issues discussed are relevant to all cadres of internationally trained health workers.

## Résumé

La migration des professionnels de la santé constitue un problème persistant en raison des inégalités qu’elle engendre. Pour y remédier, des systèmes de gouvernance mondiale axés sur la migration des professionnels de la santé ont vu le jour. Ces systèmes comprennent différents codes de pratique, accords et exigences mondiaux en matière d’établissement de rapports. Il est essentiel que ces rapports soient rigoureux, ouverts et transparents et qu’ils fassent l’objet d’un examen minutieux de la part du public, des chercheurs, des organisations de la société civile et d’autres parties prenantes intéressées. L’un des éléments de ces codes de pratiques et accords qui induit peut-être le plus grand potentiel pour faire face à l’impact de la migration des professionnels de la santé est une planification plus soutenue des professionnels de la santé afin d’atteindre l’objectif d’autosuffisance. Des plateformes ouvertes de partage de données permettent au public et aux parties prenantes d’accéder aux données sur la répartition et l’origine nationale des professionnels de la santé et révèlent les forces et faiblesses des politiques liées à la planification du personnel de santé. Dans cette étude, nous explorons les politiques récentes visant à réduire les inégalités liées à la migration des professionnels de la santé. Bien que de nombreux exemples utilisés se concentrent sur le personnel infirmier et les médecins, les questions abordées concernent également tous les supérieurs des professionnels de la santé formés à l’étranger.

## Resumen

Uno de los desafíos persistentes de la migración de los profesionales sanitarios son las desigualdades que genera. Para minimizar estas desigualdades, han surgido sistemas de gobernanza mundial de la migración de los profesionales sanitarios que incluyen diversos códigos de prácticas, acuerdos y requisitos de presentación de informes a escala mundial. Es importante que los informes sean detallados, abiertos y transparentes, y que estén sujetos al escrutinio del público, los investigadores, las organizaciones de la sociedad civil y otras partes interesadas. Uno de los elementos de estos códigos y acuerdos con mayor potencial para hacer frente al impacto de la migración de los profesionales sanitarios es una planificación más sólida del personal sanitario para alcanzar el objetivo de la autosuficiencia. Las plataformas abiertas para el intercambio de datos permiten la participación del público y las partes interesadas con datos sobre la distribución y el origen nacional de los profesionales sanitarios y revelan las fortalezas y debilidades de las políticas relacionadas con la planificación del personal sanitario. Exploramos las políticas recientes dirigidas a reducir las desigualdades derivadas de la migración de los profesionales sanitarios. Aunque muchos de los ejemplos utilizados se centran en el personal de enfermería y los médicos, los temas tratados son relevantes para todos los tipos de profesionales sanitarios con formación internacional.

## ملخص

إن التحدي المستمر الذي تواجهه هجرة العاملين في المجال الصحي يتمثل في الظروف الجائرة التي تنتج عنها. وللحد من هذه الظروف الجائرة، نشأت أنظمة الحوكمة العالمية لهجرة العاملين في المجال الصحي، والتي تشمل مدونات متنوعة للممارسات، واتفاقيات، ومتطلبات عالمية لإعداد التقارير. من المهم أن يكون إعداد التقارير صارمًا، ومنفتحًا، وشفافًا، وخاضعًا للتدقيق من الجمهور، والباحثين، ومنظمات المجتمع المدني، وغيرهم من أصحاب المصلحة المعنيين. أحد عناصر هذه المدونات والاتفاقيات، التي ربما تتمتع بأكبر قدرة لمواجهة تأثير هجرة العاملين في المجال الصحي، هو التخطيط الأكثر قوة للقوى العاملة الصحية لتحقيق هدف الاكتفاء الذاتي. تتيح المنصات المفتوحة لتبادل البيانات مشاركة الجمهور، وأصحاب المصلحة في البيانات المتعلقة بتوزيع العاملين في المجال الصحي وأصلهم الوطني، وتكشف عن نقاط القوة والضعف في سياسات تخطيط القوى العاملة الصحية. نحن نقوم باستكشاف السياسات الحديثة الموجهة نحو الحد من الظروف الجائرة الناجمة عن هجرة العاملين الصحيين. وفي حين تركز العديد من الأمثلة المستخدمة على الممرضات والأطباء، فإن القضايا التي تمت مناقشتها تتعلق بكل كوادر العاملين في المجال الصحي الذين تم تدريبهم على مستوى دولي.

## 摘要

卫生工作者移民所面临的一个持久挑战是它造成的不平等问题。为了尽量减少这些不平等现象，制定了卫生工作者移民全球管理体系，其中包括各种全球规范、协议和报告要求。提供严格、公开和透明的报告，并接受公众、研究人员、民间社会组织和其他利益相关方的监督审查，这很重要。这些规范和协议中最有可能解决卫生工作者移民造成的影响的一个要素是对卫生人力资源进行更好的规划，以实现自给自足的目标。开放的数据共享平台使公众和利益相关方能够了解有关卫生工作者分布及其原籍国的数据，并揭示了与卫生人力资源规划有关的政策优势和不足。我们探讨了近期制定的旨在减少卫生工作者移民造成的不平等现象的政策。虽然所用示例大多为护士和医生，但所讨论的问题与所有受过国际培训的骨干卫生工作者有关**。**

## Резюме

Постоянной проблемой миграции работников сферы здравоохранения является неравенство, которое она порождает. Чтобы свести к минимуму это неравенство, были созданы системы глобального управления миграцией работников сферы здравоохранения, которые включают в себя различные глобальные кодексы по практике, соглашения и требования к отчетности. Важную роль играет строгая, открытая и прозрачная отчетность, которая подлежит тщательному изучению со стороны общественности, исследователей, организаций гражданского общества и других заинтересованных сторон. Одним из важнейших элементов этих кодексов и соглашений, обладающих, возможно, наибольшим потенциалом для преодоления последствий миграции работников здравоохранения, является более тщательное планирование кадровых ресурсов здравоохранения для достижения цели по обретению самодостаточности. Открытые платформы для обмена данными позволяют привлечь общественность и заинтересованные стороны к работе с данными о распределении и национальном происхождении работников здравоохранения и выявить сильные и слабые стороны политики в сфере планирования кадровых ресурсов здравоохранения. В статье рассматриваются последние используемые меры, направленные на уменьшение неравенства в результате миграции работников сферы здравоохранения. Хотя во многих примерах речь идет о медсестрах и врачах, обсуждаемые вопросы актуальны для всех категорий работников здравоохранения, прошедших международную подготовку.

## Introduction

Health-worker migration by its nature is a global problem with local implications, given global health-workforce shortages and challenges associated with ageing populations.^1^ The transfer of health workers trained in one country to another also represents a so-called perverse subsidy in the removal of the public and private investments those workers represent.^2^ Therefore, we need to stop assessing health-workforce issues from a national perspective alone, and understand how transnational connections shape and are shaped by national policy-making on health care.^3^ International migration risks intensifying the comparative disadvantage that exists in the health systems that workers are leaving – relatively weaker workplace opportunities, pay and career advancement – thus undermining the ability of these health systems to provide universal health coverage (UHC). Assessing health-worker migration from a global perspective helps to determine its consequences for the goals of UHC and the sustainable development goals (SDGs), particularly in the wake of the coronavirus disease 2019 (COVID-19) pandemic. This issue is therefore relevant to multiple audiences, including health policy-makers, employers, managers and health workers themselves.

## Trends in health-worker migration

Health-worker migration and recruitment emerged as a global policy field in the 1950s, led by the International Labour Organization (ILO), the United Nations (UN) and the World Health Organization (WHO).^4^ The same issues of uneven development and different conditions of work remain 70 years later, but the context now reflects rapid and extensive economic globalization involving multiple global service actors, and the impact of the COVID-19 pandemic which has led to acute shortages and loss of health workers. While health-worker migration has intensified, tracking systems on the inflow and outflow of health workers can be further developed.^5^

The effects of the COVID-19 pandemic can be seen in member countries of the Organisation for Economic Co-operation and Development (OECD) where international recruitment to address health-worker shortages is evident.^6^ In the United Kingdom of Great Britain and Northern Ireland, 47% of new general practitioner trainees in 2021 were international medical graduates, and the National Health Service (NHS) aims to recruit more than 51 000 international nurses by 2024.^7^ A 2022 survey of NHS health workers revealed that 33% of doctors did not have British nationality, with the leading source countries being India (9435 doctors), Pakistan (4257), Egypt (3451) and Nigeria (2493). For nurses and health visitors, 24% (86 349/356 395) did not have British nationality, with the leading source countries being India (23 331 nurses and health visitors), Philippines (22 071), Nigeria (5537), Ireland (4419) and Zimbabwe (3380).^8^ These data do not capture health workers who have migrated but are not working in health-system roles.

In Canada, the COVID-19 pandemic has forced an already emerging health workforce crisis to intensify. Provincial governments currently engage in active recruitment efforts in the Philippines (Manitoba) and India (Newfoundland and Labrador). The federal government has also introduced policies to ease the recruitment of health workers and the integration of those workers with international qualifications already in-country.^9^ Australia’s recent push to recruit health professionals has raised concerns in New Zealand about the potential loss of workers.^10^

Since more than a million refugees entered Germany during the Syrian conflict, discussions about their integration in the labour market have included assessments of the challenges faced by health workers.^11^^,^^12^ The number of medical professionals from the Syrian Arab Republic now providing health-care services in Germany has increased to more than 5000, second after German-trained doctors.^13^

This situation has raised concerns that more migrant health workers in high-income countries will further undermine the ability of low-income countries to respond to pandemic demands.^14^ The COVID-19 pandemic has also intensified the conditions that can lead health workers to migrate, such as burn-out and poor work conditions. These trends raise the classic concerns about active recruitment from nations that overproduce health workers for the global demand, but also have national inadequate health-worker density. All countries must review international recruitment flows and determine their own self-sufficiency. Aligned with this need, all nations must address the drivers of migration, including the desire and motivations behind health-worker migration. To that end, countries need to improve working conditions and take gender into account by tackling occupational segregation of women into poorer-paid positions, the lack of women in senior leadership positions, discrimination, sexual harassment, gender bias, gender pay gap and inadequate planning for the health workforce.^15^

## Reporting health-worker migration

A global reporting structure on health-worker migration exists which is associated with various global agreements, such as WHO’s *Global code of practice on the international recruitment of health personnel*^16^ and the ILO’s *Nursing personnel convention*.^17^ However, the system has inherent challenges, including misaligned reporting functions across different data collection tools; a lack of transparency or accountability in the reporting process; and variable capacity of country respondents.^18^ Although a more coordinated approach to international requirements for reporting is needed, there is an imperative for all countries to implement more robust and transparent health-workforce planning to reduce reliance on international recruitment.

Let us take WHO’s 2010 *Global code of practice on the international recruitment of health personnel *(the Code) as an example (Box 1).^16^ The Code guides ethical recruitment and promotes investment in health systems facing shortages in the health workforce. The Code uses various provisions to achieve this goal, including reporting instruments to be completed by WHO Member States; the health workforce support and safeguard list (of countries facing critical health worker shortages); and the promotion of technical and financial assistance for low- and middle-income countries, particularly those on the safeguard list.

Box 1 **Key guiding principles in the WHO code of practice on the international recruitment of health personnel**

1. Ethical international recruitment discourages active recruitment from countries facing critical shortages.
2. Migrant health personnel should receive fair and equal treatment.
3. International cooperation between source and destination countries to derive mutual benefits is encouraged.
4. Technical assistance and financial support to developing countries is encouraged.
5. Countries are encouraged to strengthen health workforce data gathering and planning.
6. Countries should implement effective health workforce planning, development and sustainability strategies.

WHO: World Health Organization. Source: WHO, 2010.^16^

The Code includes a reporting instrument that each Member State should complete every 3 years; response rates have increased with each round. A 10-year review found the relevance of the Code was high and growing and its effectiveness had been strengthened. Nevertheless, implementation gaps remain in several countries and regions, including evidence that health ministries are not fully included in the processes related to implementation of and negotiation related to the Code and health-worker migration.^19^

The safeguard list, indicating countries with serious health-worker shortages where active recruitment should be discouraged, is dynamic. The list does not prevent voluntary migration, but suggests safeguards be in place to promote ethical recruitment, namely: upholding workers’ rights; protecting origin countries’ health systems; and encouraging destination countries to move towards self-sufficiency.^20^ Although the safeguard list offers clear direction to Member States about the most vulnerable countries, the dynamism of health-worker mobility today raises several pressing issues for source countries on and off the list.

The Philippines, for example, is often considered the leader in the export of nurses, but their reporting instrument indicates their need for more help negotiating bilateral agreements. The importance of negotiating effective bilateral agreements for health-worker mobility has been highlighted by researchers who recommend WHO works to “streamline and enhance the reporting process, gather data about and analyse the impact on the ground of these agreements, and build capacity among health ministries to not only engage but also to lead negotiations of health-worker mobility agreements.”^21^

The case of India, a prominent migration source of doctors and nurses, raises concerns about the effective protection of migrants’ rights and gender inequality related to the migration of nurses to countries in the Eastern Mediterranean region.^22^^,^^23^ Other policy decisions suggest a turning point in India’s tolerance of excess out-migration of its physicians, with the government refusing to issue so-called no obligation to return to India certificates for physicians wishing to study in the United States of America (USA), indicating that the state does not approve their leave, thus restricting their migration choices.^24^

Other examples of state actions to prevent the migration of health workers include the use of restrictive coercive policy actions. For example, reports from Zimbabwe, which is on the safeguard list, suggest the health ministry is refusing to issue nurses their certificates, effectively denying them the ability to apply for work overseas.^25^

The migration of health-care workers is a dynamic feature of the present global economy, and the different contexts we have barely revealed here clearly indicate the inequities present in the system. The good news is that structures and mechanisms are in place that encourage more effective policies that promote health-workers’ rights, protect health systems in low- and middle-income countries, and encourage greater self-sufficiency in higher-income countries to reduce their dependence on migrant health workers. The challenge is ensuring the effective use of these tools and instruments.

## Sustainable recruitment

Given the reality of increased health-worker migration before the COVID-19 pandemic and the subsequent intensification of migration, we need to ask how these practices can be made more sustainable. Improved sustainability could be achieved through ethical recruitment that embeds global health solidarity and the spirit of the Code, that is, not to undermine other nations’ health systems through recruitment. According to the 2016 *WHO Global strategy on human resources for health: workforce 2030*,^26^ Member States should aim to halve their dependence on migrant health workers by 2030. Recognizing that migration of skilled workers from low- and middle-income countries to higher-income ones will continue to happen, we would expect health-worker migration to fall more in line with the global rate of international migration which is about 3%.^27^ While migration is a normal human response to multiple factors, it should not be exploited by health systems that have failed to invest in domestic health-worker planning and training. We offer a review of select examples below to indicate what ethical and sustainable international recruitment could look like. We again consider nations within the OECD to illustrate the diversity.

Predating the Code, the Commission on graduates of foreign nursing schools international had verified the professional credentials of migrant health workers in the USA since 1977. This company also created the Alliance for ethical international recruitment practices to protect migrant health workers by encouraging ethical recruitment practices and reporting on relevant employment trends.^28^ The United States government contributed significant funds for sustainability of human resources for health in Africa through the United States President's emergency plan for AIDS relief (PEPFAR) and a renewed commitment by the Biden–Harris administration through the Global health worker initiative in 2022.^29^

In the United Kingdom, efforts have been made to promote ethical relations through mutuality or reciprocal training. The NHS’s medical training initiative allows trainees from South Asia and Africa to spend up to 2 years in an NHS training post – the learn, earn and return scheme – with the expectation that they return home.^30^ The Tropical health and education trust^31^ demonstrates leading practices in mutually beneficial health-worker training and exchange between the United Kingdom and lower-income countries. While the United Kingdom has affirmed it will adhere to the Code, its application across United Kingdom health employment sectors is voluntary.^32^

In Canada, the complex federal, provincial and territorial political system, where immigration is led federally and health care is led provincially or territorially, is a challenge to ethical recruitment.^33^ Policies focused on recruiting refugee health workers are a promising practice.^34^ The province of Nova Scotia is filling positions for older adult carers directly from refugee camps in Kenya and Jordan in partnership with UNHCR, the UN Refugee Agency, using Canada’s economic mobility pathways pilot, which identifies refugees with needed skills who meet provincial labour requirements.^35^ From an ethical standpoint this practice seems compliant, since the refugees are not providing health care in national health systems. However, the merging of refugee and economic migrant pathways raises ethical questions beyond those of health-worker recruitment. More broadly, active immigration policies create passive channels of recruitment which are not monitored from an ethical perspective.^36^

Germany’s Federal Employment Agency has recently signed bilateral agreements with India (Kerala), Indonesia and Mexico for the recruitment of nurses, which has been identified as another promising practice in line with the Code.^37^ Research on Germany’s labour immigration triple-win schemes (partnerships that benefit source and destination countries as well as migrants) in nursing have suggested success is limited by the bureaucratic nature of the schemes, suggesting further analysis is needed to determine their long-term sustainability.^38^

Together, all global health-worker agreements are about how we redistribute workers. These agreements need to be negotiated within the broader context of demographic change; that is, the acute shortage of health workers evident in countries with ageing populations, as well as internal migration dynamics that add to worker maldistribution. The demands that inform international recruitment in these situations reflect weak planning and policy-making for the health workforce. These health-worker shortages and the short-sighted planning have global ramifications that will negatively affect our ability to meet the goals of the SDGs and UHC. To improve our collective ability to manage complex health-worker migration pathways and move towards greater self-sufficiency, which will protect health systems in low- and middle-income countries, we need to develop and use data platforms, and provide technical assistance to facilitate their use across all countries.

## Need for better data

Planning helps to address the boom-bust labour market cycle of health workers and ease reliance on international recruitment of such workers. Planning should involve transparent stakeholder engagement to forge collaborative problem-solving, de-escalate political tension and overcome organizational divisions that prevent information sharing. In the Kingdom of the Netherlands, for example, an advisory committee integrates educational production and health-system needs in their workforce planning forecasts, limiting the need for international recruitment.^39^ Planning can also overcome political uncertainties by considering scenarios that include, for example, migration flows as a result of conflict. Such scenarios are increasingly likely given global instability, thus planning responses should be nested within broader policies on effective refugee integration, including refugees with health backgrounds.^12^

Planning is best supported through a strong and standardized data infrastructure. The WHO national health workforce accounts is a system through which countries can share data on their health workforce and work towards improvements in the availability, quality and use of such data. All WHO Member States have access to the national health workforce accounts’ web-based platform to upload data on country profiles, occupational profiles and key statistics. The data include health-worker density, number, age, sex, education, activity (practising, professionally active including managers, educators, researchers, and licensed to practice). Information on health facility ownership and type (public or private) is also included. Currently, the data available are insufficient for planning purposes. The platform also enables data queries on the information from the individuals with access, including place of training, where foreign training can be identified for all countries and various health professions.

The national health workforce accounts can support the gathering and sharing of standardized data on the inflow, outflow and density of health workers across source and destination country. This platform is also a comparative resource for higher-income nations to better understand the impact of recruitment of internationally educated health professionals. These data allow policy-makers and researchers to: examine country contexts; explain the health system and how it and other factors inform the drivers of international training and composition of health workers; and encourage analysis based on sustainability of the health workforce.

Inconsistencies in data reporting are evident across all countries, suggesting data collection needs to be improved. Indeed, the metadata of the national health workforce accounts show the considerable variability, periodicity, quality and completeness of the original data. These inconsistencies are to be expected in early data-system design, and highlight the importance of technical assistance to improve data collection and sharing. Since the Code encourages cooperation and technical assistance, it might be time to formalize such supports through a wealth fund where Member States contribute funds or in-kind assistance to build capacity for data collection and planning on the health workforce, especially technical support for low- and middle-income countries. This proposal echoes earlier calls for collective action to address this policy challenge.^40^ Overall, more international collaboration to support training and investment through bilateral agreements need to explicitly adopt a perspective of mutual benefits.

Better data collection and planning on the health workforce is something all Member States can embed in their national systems. This undertaking would result in overall improvements in our ability to assess and address the persistent inequities present in issues related to the health workforce, and attend more carefully to how active recruitment of health workers adds to distributional inequities.

## Ways forward

To address the heightened concerns about inequities resulting from active health-worker recruitment, we need more robust application of existing global governance tools. Recognizing that these tools – health workforce codes of practice, agreements and health-worker accounts – are both normative and voluntary, even more active support of and adherence to these tools are needed, particularly by high-income countries with greater capacity for implementation. These global tools work best when they are embedded in national health policy, planning and reporting systems, and are fully applied and supported through national policy frameworks. This reality highlights the importance of collaborative development and scale-up of technical assistance related to the health workforce. This technical assistance would facilitate both local policy, planning and decision-making and help all countries to achieve self-sufficiency in their health workforce. Furthermore, sharing technical guidance would be in keeping with the spirit of the WHO Code and other communal global governance tools.
